# A cytochrome *bd* repressed by a MarR family regulator confers resistance to metals, nitric oxide, sulfide, and cyanide in *Chromobacterium violaceum*

**DOI:** 10.1128/aem.02360-24

**Published:** 2025-01-24

**Authors:** Bianca B. Batista, Vinicius M. de Lima, W. Ryan Will, Ferric C. Fang, José F. da Silva Neto

**Affiliations:** 1 Departamento de Biologia Celular e Molecular e Bioagentes Patogênicos, Faculdade de Medicina de Ribeirão Preto, Universidade de São Paulo 54539 , Ribeirão Preto, São Paulo, Brazil; 2 Department of Laboratory Medicine and Pathology, University of Washington School of Medicine 12353 , Seattle, Washington, USA; 3 Department of Microbiology, University of Washington School of Medicine 12353 , Seattle, Washington, USA; Indiana University Bloomington, Bloomington, Indiana, USA

**Keywords:** MarR transcription factors, cytochrome *bd*, *Chromobacterium violaceum*, oxidative and nitrosative stress, metal toxicity

## Abstract

*Chromobacterium violaceum* is a ubiquitous environmental pathogen. Despite its remarkable adaptability, little is known about the mechanisms of stress resistance in this bacterium. Here, in a screen for iron-susceptible transposon mutants, we identified a cytochrome *bd* that protects *C. violaceum* against multiple stresses. The two subunits of this cytochrome *bd* (CioAB) are encoded by the *cioRAB* operon, which also encodes a GbsR-type MarR family transcription factor (CioR). A ∆*cioAB* mutant strain was sensitive to iron and the iron-requiring antibiotic streptonigrin and showed a decrease in siderophore production. Growth curves and survival assays revealed that the ∆*cioAB* strain was also sensitive to zinc, hydrogen peroxide, nitric oxide, sulfide, and cyanide. Expression analysis showed that the promoter activity of the *cioRAB* operon and the transcript levels of the *cioAB* genes were increased in a ∆*cioR* mutant. CioR bound the promoter region of the *cio* operon *in vitro*, indicating that CioR is a direct repressor of its own operon. Expression of the *cio* operon increased at high cell density and was dependent on the quorum-sensing regulator CviR. As cyanide is also a signal for *cio* expression, and production of endogenous cyanide is known to be a quorum sensing-regulated trait in *C. violaceum*, we suggest that CioAB is a cyanide-insensitive terminal oxidase that allows respiration under cyanogenic growth conditions. Our findings indicate that the cytochrome *bd* CioAB protects *C. violaceum* against multiple stress agents that are potentially produced endogenously or during interactions with a host.

**IMPORTANCE:**

The terminal oxidases of bacterial respiratory chains rely on heme-copper (heme-copper oxidases) or heme (cytochrome *bd*) to catalyze the reduction of molecular oxygen to water. *Chromobacterium violaceum* is a facultative anaerobic bacterium that uses oxygen and other electron acceptors for respiration under conditions of varying oxygen availability. The *C. violaceum* genome encodes multiple respiratory terminal oxidases, but their role and regulation remain unexplored. Here, we demonstrate that CioAB, the single cytochrome *bd* from *C. violaceum*, protects this bacterium against multiple stressors that are inhibitors of heme-copper oxidases, including nitric oxide, sulfide, and cyanide. CioAB also confers *C. violaceum* resistance to iron, zinc, and hydrogen peroxide. This cytochrome *bd* is encoded by the *cioRAB* operon, which is under direct repression by the MarR-type regulator CioR. In addition, the *cioRAB* operon responds to quorum sensing and to cyanide, suggesting a protective mechanism of increasing CioAB in the setting of high endogenous cyanide production.

## INTRODUCTION

Many bacteria have flexible and branched respiratory chains that allow them to obtain energy through aerobic and anaerobic respiration under diverse conditions (1). Terminal respiratory oxygen reductases are membrane-integrated enzymes that use an electron donor (cytochrome *c* or quinol) to catalyze the reduction of molecular oxygen to water at the end of aerobic respiratory chains. These enzymes belong to the evolutionary unrelated superfamilies of heme-copper oxidases (HCOs), alternative oxidases, and cytochrome *bd*-type oxygen reductases (cytbd) (2–4). While the HCO superfamily is widespread from bacteria to eukaryotic mitochondria, the cytbd superfamily is restricted to bacteria and archaea (3, 4).

Most redox reactions catalyzed by respiratory enzymes involve metal centers containing iron (as heme and iron-sulfur clusters) and copper. The HCO enzymes use a catalytic heme-copper binuclear center. In contrast, the cytbd enzymes contain heme but not copper ions (3). The cytochrome *bd* oxygen reductases are quinol oxidases composed of two main integral membrane proteins, subunits I (CydA) and II (CydB), and an auxiliary small subunit (CydX) (cytochrome *bd*-I from *Escherichia coli*). CydA contains three heme groups (*b*558, *b*595, and *d*) and a quinol-binding site. A hydrophobic region of CydA involved in quinol oxidation, called the Q-loop, has variable length. Based on this feature, cytbd reductases have been divided into two groups: L (long Q-loop) and S (short Q-loop). A third group includes cytochrome *bd* containing only b hemes, called cyanide-insensitive terminal oxidases (CIO) (2, 5, 6).

Cytochrome *bd* protects bacteria against several environmental or host-derived stressors, including nitrosative and oxidative stress and metal toxicity (7–12). Accordingly, in some bacteria, cytochrome *bd* mutants are severely compromised in virulence and intracellular viability (13–15). Cytochrome *bd* also protects bacteria from antibacterial drugs and are attractive targets for new antibiotics (3, 16–18). The expression of cytochrome *bd* involves different regulatory systems. In *E. coli*, the *cydAB* genes reach maximum expression in microaerobic conditions by the concerted actions of ArcAB and Fnr, two oxygen-responsive regulatory systems (19, 20). In *Alishewanella* sp., the *cydAB* genes are repressed by CydE, a GbsR-type regulator (21). The GbsR proteins comprise a poorly characterized subfamily of regulators belonging to the MarR (multiple antibiotic resistance regulator) family of transcription factors. Genes encoding GbsR-like regulators are clustered in bacterial genomes with genes encoding cytochrome *bd* or transporters for osmoprotection (21, 22).

*Chromobacterium violaceum* is a facultative anaerobic Gram-negative bacterium commonly isolated from soil and water that causes rare but deadly infections in humans and other animals (23, 24). Many *C. violaceum* traits such as production of violacein, exoenzymes, and cyanide are regulated by the LuxR-type quorum sensing (QS) system CviI/CviR (25, 26). Although many species of the *Chromobacterium* genus are producers of cyanide (25, 27–29), it is unclear how these bacteria protect themselves from cyanide toxicity. *C. violaceum* has versatile respiratory capacity, exhibiting both cyanide-sensitive and -insensitive aerobic respiratory activities (30) and anaerobic nitrate respiration (31). Genes encoding both HCO and cytbd-type respiratory terminal oxidases are predicted in the *C. violaceum* genome, but their role and regulation remain unexplored. In this work, we demonstrated that a cytochrome *bd* (CioAB) repressed by a MarR-type transcription factor (CioR) protects *C. violaceum* against multiple inhibitors of HCO oxidases, including the poison cyanide.

## RESULTS

### Cytochrome *bd* encoded by the *cioRAB* operon protects *C. violaceum* from iron toxicity

**Fig 1:**
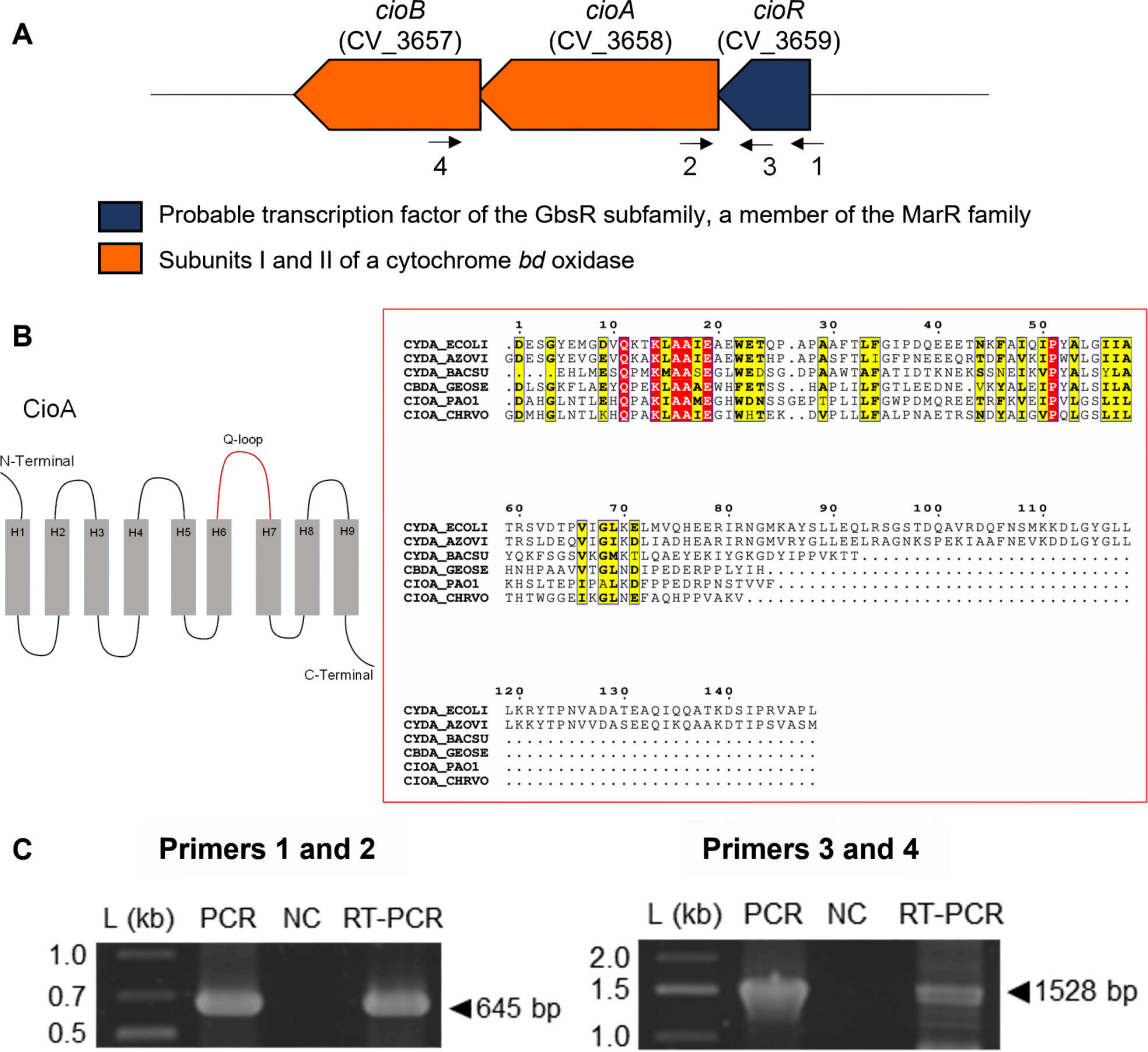
The *cioRAB* genes comprise an operon encoding a MarR-type regulator and a cytochrome *bd*. (A) Genomic organization of the *cioRAB* genes in *C. violaceum*. Numbered arrows indicate primers used in RT-PCR. (B) CioAB is a cytochrome *bd* with a short Q-loop in CioA. The diagram represents the membrane topology of CioA from *C. violaceum*. CioA has nine transmembrane segments (H1 to H9). The Q-loop involved in quinol oxidation is highlighted in red. Multiple alignment of the Q-loop region from *C. violaceum* CioA with selected orthologs is shown. Proteins are from *C. violaceum* (CHRVO), *Escherichia coli* (ECOLI), *Azotobacter vinelandii* (AZOVI), *Bacillus subtilis* (BACSU), *Geobacillus stearothermophilus* (GEOSE), and *Pseudomonas aeruginosa* (PAO1). Identical and similar residues are boxed in red and yellow, respectively. (C) Co-transcription of the *cioRAB* genes. The RT-PCR reactions amplified fragments of 645 bp (Primers 1 and 2) and 1528 bp (Primers 3 and 4) using RNA from WT *C. violaceum*. Conventional PCR was performed using genomic DNA (PCR) and RNA (NC), as controls. L, 1 Kb plus DNA Ladder (Thermo Scientific).

**Fig 2:**
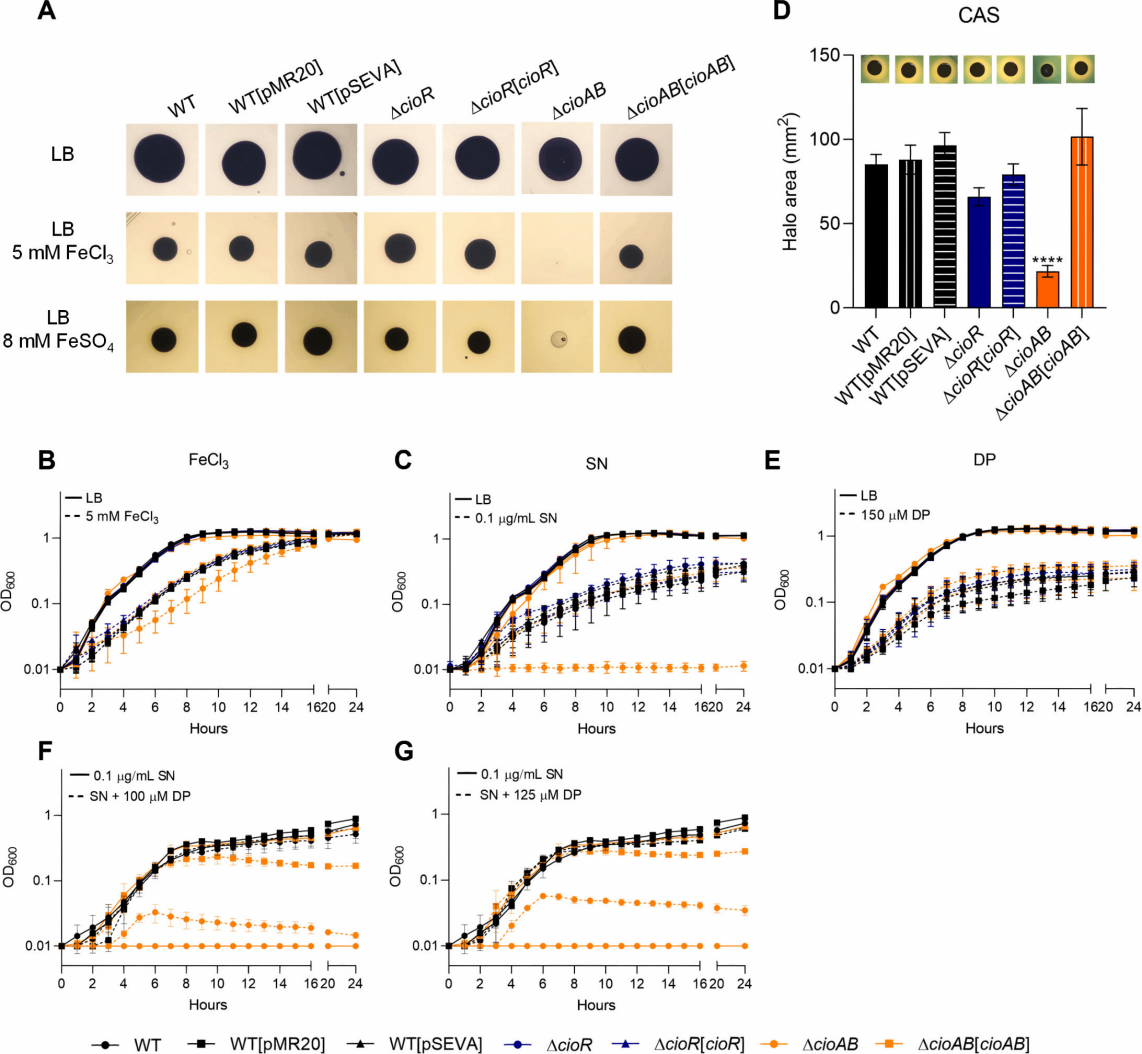
The cytochrome *bd* CioAB protects *C. violaceum* against iron toxicity. (A) Iron susceptibility plate assays. The indicated strains were grown for 24 h at 37°C on LB plates without or with 5 mM FeCl_3_ or 8 mM FeSO_4_. (B, C, E, F, G) Growth curves were obtained on Bioscreen C or BioTek Epoch2 microplate readers. The indicated strains were grown in LB medium in 96-well plates for 24 h at 37°C under agitation without (continuous lines) or with (dashed lines) the indicated treatments. (B) 5 mM ferric chloride (FeCl_3_). (C) 0.1 µg/mL streptonigrin (SN). (E) 150 µM 2,2′-dipyridyl (DP). (F) 0.1 µg/mL streptonigrin (SN) plus 100 µM 2,2′-dipyridyl (DP). (G) 0.1 µg/mL streptonigrin (SN) plus 125 µM 2,2′-dipyridyl (DP). (D) Mutation of *cioAB* leads to decreased siderophore halos. Measurement of the CAS halo diameter of the indicated strains. Data from three biological assays. Insert: illustrative PSA-CAS plates showing siderophore production (orange halos). ^****^P < 0.0001; when not indicated, not significant. One-way ANOVA followed by Tukey’s multiple-comparison test.

We screened a *C. violaceum* transposon mutant library (26) for iron-susceptible transposon mutants on LB plates with 5 mM FeCl_3_ or 8 mM FeSO_4_. Mutant strains with impaired growth in iron excess had transposon insertions in genes related to regulatory systems and sugar metabolism (Table S1). One such mutant, with a transposon inserted in the gene CV_3659 (Table S1), was further investigated in this work. This gene, here named *cioR*, encodes a putative transcriptional regulator belonging to the GbsR subgroup of MarR family transcription factors (21, 22). The *cioR* gene is located adjacent to the genes CV_3658 and CV_3657, which encode the subunits I (CioA) and II (CioB) of a cytochrome *bd* quinol oxidase (Fig. 1A). *C. violaceum* CioA presents a short Q-loop (Fig. 1B) as in CioA from *Pseudomonas aeruginosa* (44.3% identity), CbdA from *Geobacillus stearothermophilus* (29.9% identity), and CydA from *Bacillus subtilis* (33% identity). RT-PCR reactions using RNA from wild-type (WT) *C. violaceum* and primers corresponding to regions between the genes *cioR-cioA* and *cioR-cioB* amplified DNA bands with expected sizes (Fig. 1A and C), indicating that these genes are co-transcribed, comprising the *cioRAB* operon.

ince a *cioR* transposon mutant was impaired for growth at high iron concentrations (Table S1), we constructed null mutant strains to test the role of the *cioRAB* operon in iron tolerance (Fig. 2). A ∆*cioR* mutant strain had no growth impairment in the iron susceptibility plate assays used in the transposon screen, whereas a ∆*cioAB* mutant failed to grow under the identical condition (Fig. 2A). This suggested that the iron-susceptible phenotype of the *cioR* transposon mutant was due to a polar effect on the *cioAB* genes. Indeed, ∆*cioAB* but not ∆*cioR* strains exhibited increased sensitivity to iron and the iron-requiring antibiotic streptonigrin (SN) in growth curves (Fig. 2B and C) and decreased production of siderophores on peptone-sucrose agar with Chrome Azurol S (PSA-CAS) plates (Fig. 2D). All mutant strains grew similarly to WT under iron-deficient conditions (treatment with 2,2′-dipyridyl, DP) (Fig. 2E). A Δ*cioAB* mutant showed a modest but reproducible growth improvement with SN in the presence of increasing DP concentrations (Fig. 2F and G). This partial growth reversion suggests that while the role of iron is important, it is not sufficient to explain the high sensitivity of Δ*cioAB* to SN. The sensitivity of a ∆*cioAB* mutant to FeCl_3_ and SN was further confirmed by survival and disk diffusion assays (Fig. S1A and B). All of these iron-related phenotypes were reversed by complementation of the ∆*cioAB* strain (Fig. 2; Fig. S1A and B). Collectively, these data indicate that the cytochrome *bd* CioAB protects *C. violaceum* against iron toxicity. Quantification by inductively coupled plasma mass spectrometry demonstrated similar iron levels in the WT, Δ*cioR*, and Δ*cioAB* strains (data not shown), suggesting that the SN and siderophore phenotypes of a Δ*cioAB* mutant could be related to high levels of free intracellular iron (in the ferrous form) rather than a difference in total iron quantity. In *E. coli, cyd* mutants (*bd*-deficient) exhibit reduced iron content and increased siderophore production (32).

### The cytochrome *bd* CioAB confers resistance of *C. violaceum* to zinc, hydrogen peroxide, nitric oxide, sulfide, and cyanide

**Fig 3:**
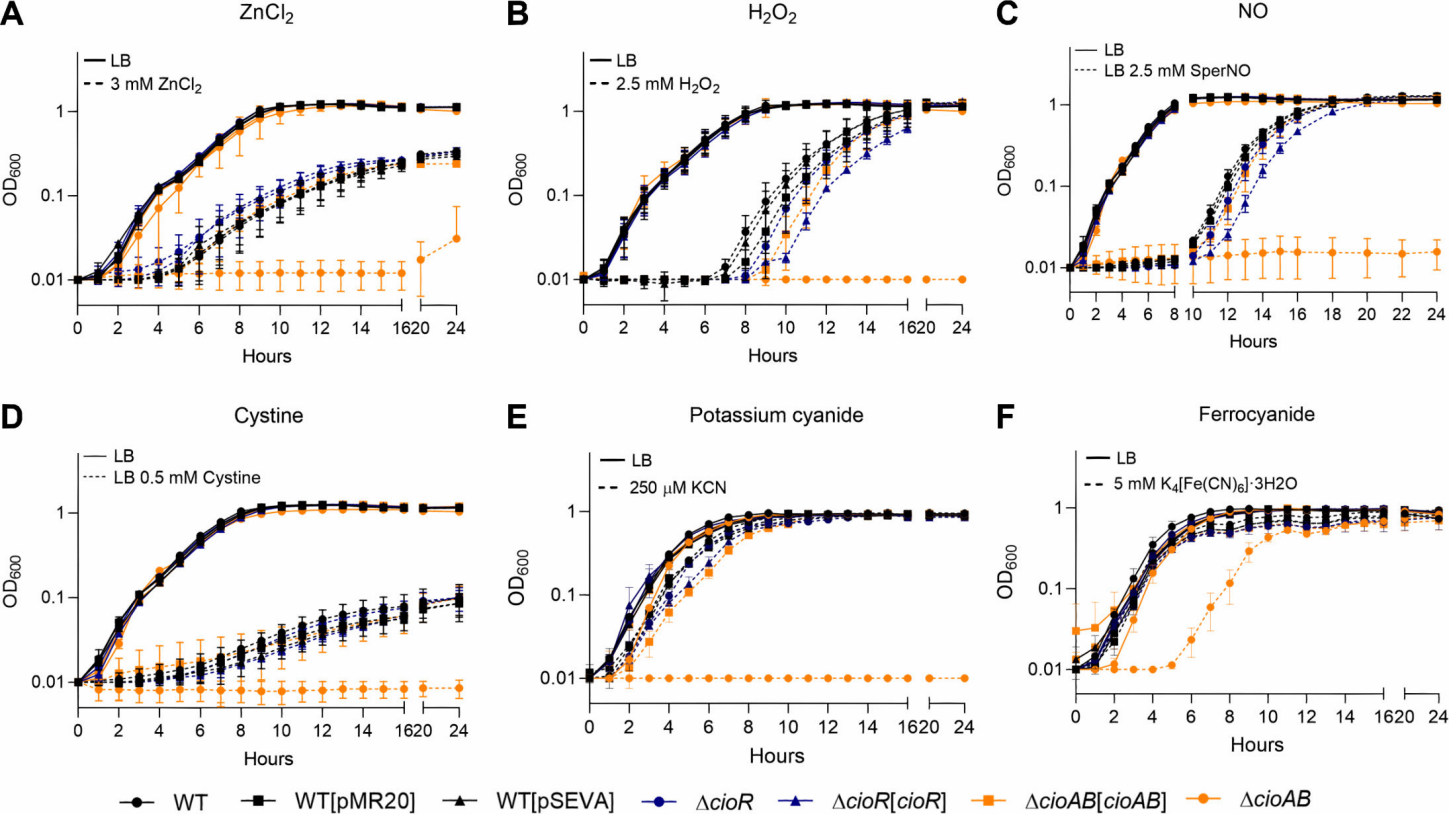
The cytochrome *bd* CioAB protects *C. violaceum* against toxicity of metal, oxidative, nitrosative, sulfide, and cyanide stresses. (A–F) Growth curves were obtained on Bioscreen C or BioTek Epoch2 microplate readers. The indicated strains were grown in LB medium in 100-well or 96-well plates for 24 h at 37°C under agitation without (continuous lines) or with (dashed lines) the indicated treatments. (A) 3 mM zinc chloride (ZnCl_2_). (B) 2.5 mM hydrogen peroxide (H_2_O_2_). (C) 2.5 mM spermine NONOate (SperNO, NO donor). (D) 0.5 mM cystine (sulfide production). (E) 250 µM potassium cyanide (KCN). (F) 5 mM potassium ferrocyanide (K_4_[Fe(CN)_6_]·3H_2_O).

The importance of the *cioRAB* operon in protecting *C. violaceum* under other stress conditions was evaluated by growth curves comparing the WT strain with the *cio* mutants and complemented mutant strains (Fig. 3). A Δ*cioAB* mutant was more susceptible to zinc (ZnCl_2_), hydrogen peroxide (H_2_O_2_), nitric oxide (sperNO), sulfide (cystine), potassium cyanide (KCN), and potassium ferrocyanide (K_4_[Fe(CN)_6_]·3H_2_O), compared with the WT and complemented strains (Fig. 3). A ∆*cioR* mutant exhibited no growth defect under each of the tested stress conditions (Fig. 3). Sensitivity of the ∆*cioAB* mutant to ZnCl_2_ and H_2_O_2_ was further confirmed by survival and disk diffusion assays (Fig. S1A and B). These data indicate that *C. violaceum* relies on cytochrome *bd* CioAB to grow and survive in the presence of diverse toxic compounds. We hypothesized that the mechanism of stress resistance might involve the tolerance of CioAB to inhibition by these stressors, as many of them, including zinc, nitric oxide, sulfide, and cyanide, are known to inhibit HCO but not cytbd respiratory terminal oxidases in other bacteria (5, 8, 33, 34). The marked sensitivity of a Δ*cioAB* mutant to cyanide (Fig. 3E) indicates that expression of CioAB is a key mechanism for *C. violaceum* resistance to this compound, which is endogenously produced by many *Chromobacterium* species (25, 27–29). WT *C. violaceum* was highly tolerant to cyanide, growing in concentrations up to mM of exogenously added potassium cyanide (Fig. S1C). Curiously, when growth curves in LB were performed in large volumes (glass tubes instead of microplates), a Δ*cioAB* mutant reached a lower cell density after the late-exponential growth phase even in the absence of any exogenous stress (Fig. S1D). This growth impairment of the Δ*cioAB* mutant in LB was also observed in survival assays of stationary growth phase cultures (Fig. S1A). As cyanide accumulates from late-exponential growth phase in cultures of *C. violaceum* and other cyanogenic *Chromobacterium* species (27–30), it is conceivable that the growth of a Δ*cioAB* mutant might be affected by endogenous cyanide. While our data clearly support the role of CioAB in protecting against exogenous cyanide, its contribution as a defense mechanism against cyanide autointoxication remains to be further explored.

### The *cioRAB* operon is repressed by CioR and activated by the quorum-sensing regulator CviR

**Fig 4:**
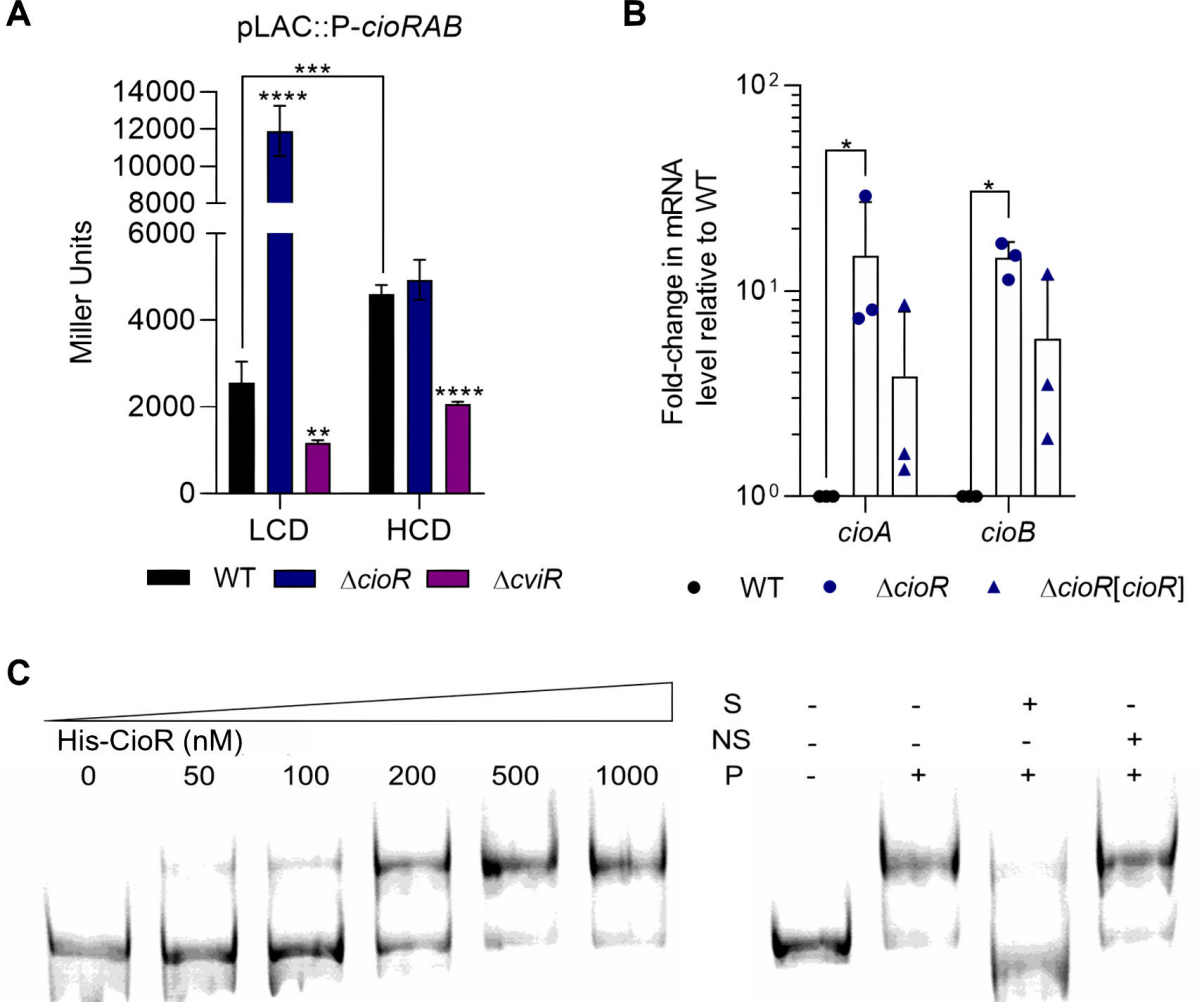
The *cioRAB* operon is directly repressed by CioR and activated by the QS regulator CviR. (A) Expression of the *cioRAB* operon varies according to cell density and is regulated by CioR and CviR. Promoter activity was measured by β-galactosidase assays performed from WT, ∆*cioR*, and ∆*cviR* strains harboring the *cioRAB-lacZ* fusion. Strains were grown in LB medium in low cell density (LCD, OD_600_ 1.0) or high cell density (HCD, OD_600_ 4.0). Data are from five biological replicates. ^**^P < 0.01; ^***^P < 0.001; ^****^P < 0.0001; when not indicated, not significant. Two-way ANOVA followed by Sidak’s multiple comparisons test. (B) The *cioAB* genes are repressed by CioR. RNA obtained from WT, ∆*cioR*, and ∆*cioR*[*cioR*] strains grown in LB until OD_600_ 1.0 was reverse transcribed to cDNA and used for RT-qPCR reactions. Expression of *cioA* and *cioB* is shown as fold-change relative to the control condition (WT). Data are from three biological replicates. ^*^P < 0.05; when not indicated, not significant. Two-way ANOVA followed by Tukey’s multiple-comparison test. (C) CioR binds to the promoter region of the *cioRAB* operon. The indicated concentrations of His-CioR were used in EMSA with a *cioRAB*-FAM probe. Competition assays with unlabeled probes were performed to check binding specificity. S, specific unlabeled probe; NS, nonspecific unlabeled probe; P- 1 µM His-CioR protein.

CioR is predicted to be a GbsR-type transcriptional regulator on the basis of sequence homology (Fig. 1A). Because CioAB protects *C. violaceum* against cyanide (Fig. 3E), and production of this compound relies on the QS system CviI/CviR (25, 29), we tested the effect of QS and CioR on the expression of the *cioRAB* operon. The promoter region of the *cioRAB* operon was fused with the *lacZ* gene, and the plasmid-based reporter construction was used to measure its expression at low (LCD) and high (HCD) cell density, comparing expression between WT and mutant strains (Fig. 4A). Beta-galactosidase assays with the WT strain showed that the *cioRAB* operon has a strong promoter that is upregulated from LCD to HCD. Mutation of *cioR* increased the expression of the *cioRAB* operon at LCD (Fig. 4A). Expression of the *cioRAB* operon decreased in a Δ*cviR* mutant compared to WT under both cell density conditions (Fig. 4A). These data indicate that the *cioRAB* operon is repressed by CioR at LCD and activated by CviR at LCD and HCD. To further demonstrate the role of CioR as a repressor of its own operon, we quantified *cioA* and *cioB* transcripts in the WT and Δ*cioR* strains by RT-qPCR. As expected, expression of both genes was increased in a Δ*cioR* mutant compared to WT and complemented strains (Fig. 4B). To verify that CioR is a direct repressor of its own operon, we performed electrophoretic mobility shift assays (EMSA). The purified His-CioR protein was able to specifically bind to a DNA probe containing the promoter region of the *cioRAB* operon (Fig. 4C), confirming its direct regulation by CioR.

### Expression of the *cioRAB* operon increases after exposure to exogenous cyanide

**Fig 5:**
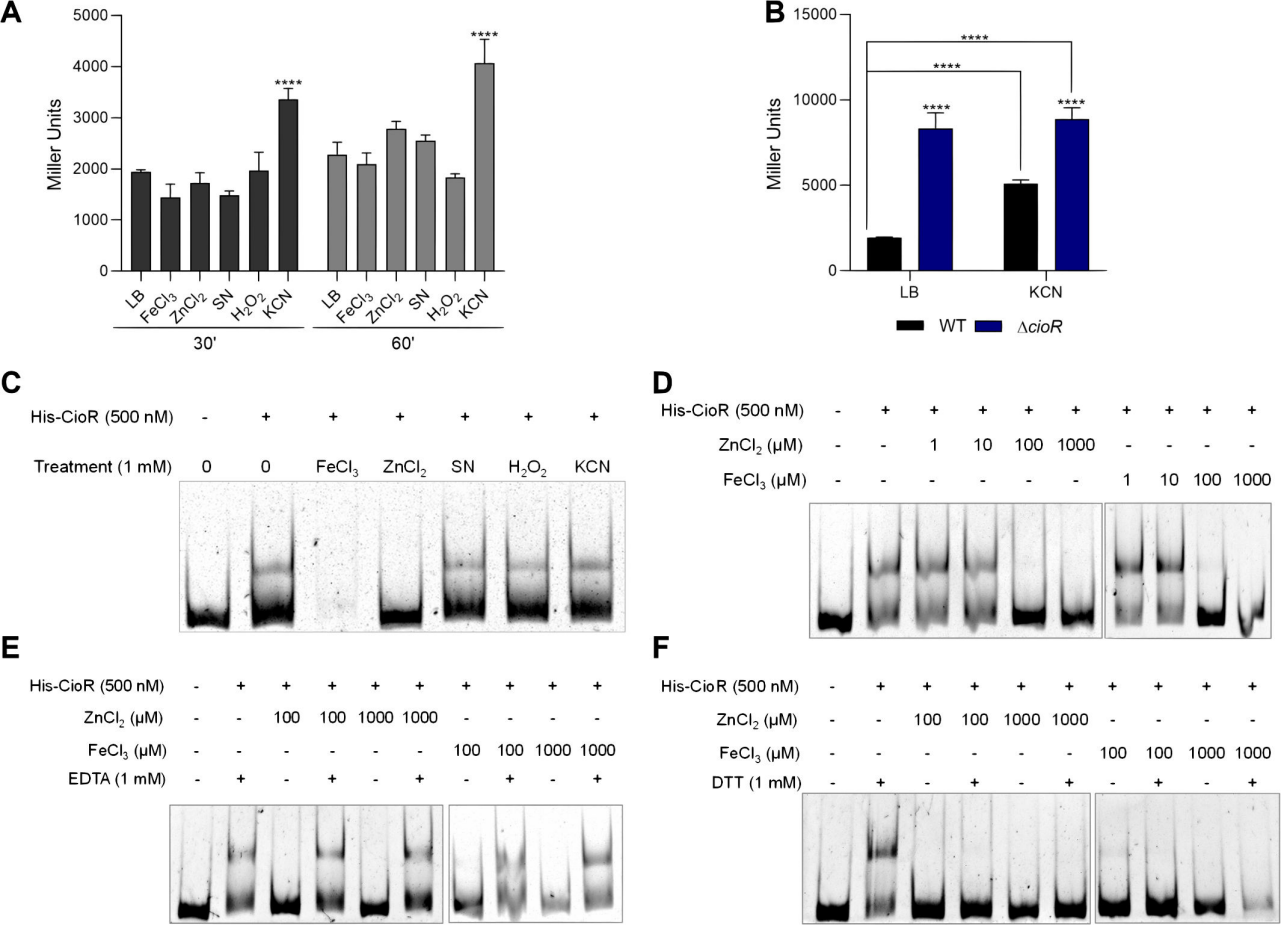
The *cioRAB* operon is induced in the presence of cyanide. (A) Promoter activity of the *cioRAB* operon under treatment with different stress agents. For the β-galactosidase assays, cultures of the WT strain harboring the *cioRAB-lacZ* fusion were grown in LB medium until OD_600_ 0.5 and untreated or treated for 30 and 60 min with the following compounds: FeCl_3_, 5 mM ferric chloride; ZnCl_2_, 2 mM zinc chloride; SN, 0.5 µg/mL streptonigrin; H_2_O_2_, 10 mM hydrogen peroxide; KCN, 250 µM cyanide. (B) Promoter activity of the *cioRAB* operon in the indicated strains. Cultures were grown in LB medium until OD_600_ 0.5 and untreated or treated for 60 min with 250 µM KCN. Data are from three biological replicates. ^****^P < 0.0001; when not indicated, not significant. Two-way ANOVA followed by Sidak’s multiple comparisons test. (C–F) Zinc and iron disrupt CioR DNA binding. The purified His-CioR was used in EMSA with a *cioRAB* unlabeled probe. The indicated compounds were added in the reactions. Detection by ethidium bromide staining.

Because CioAB confers *C. violaceum* resistance to multiple stress conditions (Fig. 2; Fig. 3), we measured the expression of the *cioRAB* operon in response to stressors, using the WT strain carrying a p*lacZ*::P*cioRAB* fusion (Fig. 5A). Five distinct compounds were individually added to cultures of WT *C. violaceum* in LB at LCD for 30 and 60 min. Under these conditions, the expression of the *cioRAB* operon increased only with cyanide at both time points compared to an untreated LB control (Fig. 5A). These data indicate that, of the tested toxic compounds to which CioAB confers protection, only cyanide acts as an inducing signal for the *cioRAB* operon. To test the role of CioR in this cyanide-dependent expression, we performed these assays in a ∆*cioR* mutant. In this mutant background, the *cioRAB* expression was similarly high both with and without cyanide (Fig. 5B).

In addition, we evaluated by EMSA whether cyanide or other stressors were capable of inactivating CioR *in vitro*. At a high concentration (1 mM), iron and zinc, but not KCN, SN, or H_2_O_2_, abrogated the binding of CioR to the DNA probe containing the *cio* promoter (Fig. 5C). By testing other concentrations of iron and zinc (1, 10, 100, and 1000 µM), we found that 100 µM was sufficient for both metals to disrupt CioR binding to DNA (Fig. 5D). These effects were reversed by adding the metal chelator EDTA (Fig. 5E) but not the reducing agent DTT (Fig. 5F). These data suggest that although CioR contains a Cys residue (C52), its metal inactivation likely involves other metal-binding amino acids. Indeed, analysis using AlphaFold predicted that zinc and iron bind to CioR at histidine residues (data not shown). In *E. coli*, the transcriptional regulator MarR is inactivated by copper via cysteine oxidation (35). Based on our data, we suggest that CioR is not a direct cyanide sensor.

## DISCUSSION

In this work, we demonstrated the regulation of the *cioRAB* operon by the GbsR-type regulator CioR and showed that a key role of the cytochrome *bd* CioAB is to protect *C. violaceum* against multiple environmental insults, including the poison cyanide and the antimicrobial molecule nitric oxide. Our findings further indicate that activation of the *cioRAB* operon by the quorum-sensing regulator CviR provides a regulatory mechanism to maximize CioAB expression at HCD, a condition in which *C. violaceum* is known to increase endogenous cyanide production.

Our screen of a *C. violaceum* transposon mutant library revealed iron-susceptible mutants with transposon insertions in genes related to gene regulation and in a gene cluster for the synthesis of polysaccharide precursors. A transposon screen in *Xanthomonas campestris* identified a β-(1,2)-glucan that protects against iron toxicity (36). A transposon insertion was found in *cioR*, but phenotypic characterization of Δ*cioR* and Δ*cioAB* null mutants revealed that deletion of *cioAB* but not *cioR* enhanced *C. violaceum* susceptibility to iron, zinc, hydrogen peroxide, nitric oxide, sulfide, and cyanide. The protective role of CioAB for many of these stressors is likely to involve tolerance of cytochrome *bd* to inhibition by compounds that are potent inhibitors of HCO terminal oxidases, including zinc (33), sulfide (34), nitric oxide (NO) (8), and cyanide (5). Catalase activity of cytochrome *bd* accounts for hydrogen peroxide resistance (9, 10, 37), and a rapid dissociation rate accounts for its insensitivity to nitric oxide (8). While cytochrome *bd* mutations result in iron deprivation in other bacteria (7, 32), a *C. violaceum* Δ*cioAB* mutant exhibited enhanced susceptibility to SN and decreased siderophore production, but an unaltered iron content, consistent with increased free intracellular Fe(II). Previous work has shown that non-respiring *E. coli* accumulates NADH, which accelerates flavin reductase-mediated electron transfer to free flavins, which in turn can reduce free iron to catalyze the Fenton reaction and promote DNA damage (38). As SN depends on Fe(II) for its killing activity (39), we suggest that a similar mechanism could account for the iron, H_2_O_2_, and SN susceptibility of a *C. violaceum* Δ*cioAB* mutant. Increased Fe(II) could also account for the decreased siderophore production via Fur-mediated repression of genes related to siderophore biosynthesis (40).

In this work, we have identified different conditions and transcriptional regulators controlling the expression of the *cioRAB* operon in *C. violaceum*. We found that CioR, belonging to the GbsR subfamily of MarR family transcription factors (21, 22), acts as a direct repressor of its own operon. The only previously characterized GbsR-type regulator associated with cytochrome *bd* genes, CydE, represses the *cydEAB* operon of *Alishewanella* and appears to respond to sulfate (21). In both cases, this operon organization places the expression of the cytbd within a feedback circuit of autorepression that has many regulatory implications, such as limiting the duration of promoter activation. In *C. violaceum*, we found that, among several stressors, exogenous cyanide induces the *cioRAB* operon in a CioR-dependent manner. However, CioR does not appear to be a direct cyanide sensor. Indeed, indirect sensing has been described for other cyanide-responsive regulators. The MpaR regulator responds to cyanide-modified pyridoxal phosphate molecules in *P. aeruginosa* (41), and TstR senses sulfite and Fe(III) to control cyanide resistance in *Lactobacillus brevis* (42). Based on our data, we suggest that cyanide induces the *cioRAB* operon by indirectly increasing, via inactivation of metalloenzymes, the intracellular availability of zinc or iron, which in turn inactivates the repressor CioR.

We have previously demonstrated that the MarR family regulators OhrR and OsbR coordinate responses against oxidative stress in *C. violaceum* (31, 43, 44). OsbR has a large regulon, including the *nar* genes involved in anaerobic nitrate respiration and the *cioRAB* operon (31). Interestingly, in addition to direct repression by OsbR (31) and CioR, we have shown here that the *cioRAB* operon is also regulated by cell density and activated by the QS regulator CviR, a result that is consistent with a recent global transcriptome analysis (26). As OsbR and CioR were activated by CviR (26), and both transcription factors act as direct repressors of the *cioRAB* operon, it is possible that the activation of the *cio* operon via CviR could be due to other regulatory cascades or direct CviR antagonism of these repressors. Given that cyanide production is also a QS-regulated process (25, 29) employed by *Chromobacterium* species for predation evasion (27), bacterial competition (29), and larval killing (28), our data demonstrating the marked susceptibility of a Δ*cioRAB* mutant to cyanide suggest that cyanide tolerance conferred by the cytochrome *bd* CioAB is likely to play an important role in many facets of *C. violaceum* biology. In *P. aeruginosa*, QS and cyanide regulation of *cioAB* contributes to cooperative behavior in bacterial populations (45).

Many bacterial pathogens require cytochrome *bd* enzymes during host infection, including *Vibrio cholerae* (14), *Salmonella enterica* serovar Typhimurium (12), *Mycobacterium tuberculosis* (46), uropathogenic *Escherichia coli* (15), and *Listeria monocytogenes* (47). In some cases, the role in virulence involves cytochrome *bd*-mediated protection against nitric oxide (NO) released by host cells (12, 15). Recently, macrophage-produced NO has been implicated in controlling *C. violaceum* infection in the mouse liver within granulomas (48). Additional studies will be required to elucidate the specific role of the cytochrome *bd* CioAB in *C. violaceum* virulence, given its protective role against multiple stress conditions including cyanide and nitrosative stress.

## MATERIALS AND METHODS

### Bacterial strains, plasmids, and growth conditions

Bacterial strains and plasmids used in this work are described in Table 1. *E. coli* and *C. violaceum* strains were cultured in Luria-Bertani (LB) medium. When appropriate, cultures were supplemented with kanamycin (50 µg/mL), ampicillin (100 µg/mL), or tetracycline (10 µg/mL).

**TABLE 1:** Strains and plasmids

Strain or plasmid	Description^*a*^	Reference or source
Strains		
*Escherichia coli*		
DH5α	*E. coli* strain for cloning purposes	(49)
S17-1	*E. coli* strain for plasmid mobilization	(50)
BL21(DE3)	*E. coli* strain for heterologous expression of proteins	Novagen
*Chromobacterium violaceum*		
ATCC 12472 (WT)	*C. violaceum* ATCC 12472 wild-type (WT) strain with sequenced reference genome	(51)
CV^NALR^	Strain with *gyrA* spontaneous mutation, for transposon mutant selection	(40)
WT[pMR20]	WT control strain harboring the empty pMR20 plasmid	This work
WT[pSEVA]	WT control strain harboring the empty pSEVA plasmid	This work
WT[pLAC::P*cioRAB*]	WT strain with the *cioRAB*-l*acZ* fusion	This work
∆*cioR*	WT strain with *cioR* (CV_3659) gene deleted	This work
∆*cioR*[*cioR*]	Δ*cioR* mutant complemented with WT copy of *cioR* in the pSEVA vector	This work
∆*cioR*[pLAC::P*cioRAB*]	Δ*cioR* strain with the *cioRAB*-l*acZ* fusion	This work
∆*cioAB*	WT strain with *cioAB* (CV_3657 and CV_3658) genes deleted	This work
∆*cioAB*[*cioAB*]	Δ*cioAB* mutant complemented with WT copy of *cioAB* in the pMR20 vector	This work
∆*cviR*	WT strain with *cviR* (CV_4090) gene deleted	(52)
∆*cviR*[pLAC::P*cioRAB*]	Δ*cviR* strain with the *cioRAB*-l*acZ* fusion	This work
Plasmids		
pNPTS138	Suicide vector containing oriT, *sacB*; Kan^R^	M.R.K. Alley
pMR20	Broad-host-range low-copy vector containing oriT, Tet^R^	(53)
pSEVA221	Broad-host-range low-copy vector, Kan^R^	(54)
pET15b	Expression of proteins with N-terminal His-tag; Amp^R^	Novagem
pGEM-T easy	Cloning plasmid; Amp^R^	Promega
pRK*lacZ*290	pRK2-derived vector with promoterless *lacZ* gene, Tet^R^	(55)

^*a*^ Kan, kanamycin; Tet, tetracycline; Amp, ampicillin; NAL, nalidixic acid; R, resistance.

### *In silico* analysis of CioA

Protein sequences of cytochrome *bd* subunit I from *C. violaceum* (CioA), *E. coli* (CydA), *A. vinelandii* (CydA)*, B. subtilis* (CydA), *G. stearothermophilus* (CbdA)*,* and *P. aeruginosa* (CioA) were retrieved from UniProt (https://www.uniprot.org/). Transmembrane helices were predicted using TMHMM - 2.0 (https://services.healthtech.dtu.dk/services/TMHMM-2.0/). Amino acids of the Q-loop region (located between the transmembrane helices six and seven) were selected for multiple sequence alignment using Clustal Omega (https://www.ebi.ac.uk/Tools/msa/clustalo/) and visualization with ESPript 3.x (https://espript.ibcp.fr/ESPript/ESPript/).

### Screening of a transposon mutant library for iron toxicity

A previously obtained *C. violaceum* 10,000-mutant library (26) was screened for iron susceptibility by spotting 10 µL of overnight cultures of individual clones on LB plates supplemented or not with 5 mM FeCl_3_ or 8 mM FeSO_4_. Transposon mutant strains with growth impairment after incubation for 24 h at 37°C under iron excess were selected for identification of transposon insertion sites by semi-degenerate PCR (Table S2), followed by Sanger sequencing as previously described (26, 40, 56). Null *cio* mutants were also tested in the iron-supplemented plate assays.

### Construction of *C. violaceum* mutant and complemented strains

All null-mutant strains were derived from the *C. violaceum* wild-type strain ATCC 12472. In-frame null deletions of *cioR* (∆*cioR*) and *cioAB* (∆*cioAB*) were generated by allelic exchange mutagenesis using sucrose for counterselection, as previously described (26, 40, 43, 57). Null mutant strains were complemented by providing wild-type copies of the mutated genes cloned into pMR20 (for *cioAB*) or pSEVA (for *cioR*) plasmids. Primers used for cloning, sequencing, and confirmation of mutant and complemented strains are listed in Table S2.

### Growth curves

*C. violaceum* wild-type, mutant, and complemented strains were grown in LB medium overnight. Cultures were diluted to an optical density at 600 nm (OD_600_) of 0.01 in LB that was untreated or treated with individual or combined stress agents to a final volume of 150 µL in 96-well or 300 µL in 100-well microplates. Cultures were grown aerobically with shaking at 37°C in Bioscreen C or BioTek Epoch2 microplate readers. Growth was monitored by measuring OD_600_ every 60 min. Experiments were performed with six replicates. Treatment conditions are indicated in the respective figures. A set of growth curves in large volumes (5 mL cultures) were obtained in glass tubes containing LB that were incubated at 37°C under agitation. Aliquots were taken to measure OD_600_ in an Eppendorf BioPhotometer.

### Survival assays

*C. violaceum* wild-type, mutant, or complemented strains were grown in LB overnight. Cultures were diluted to OD_600_ 0.01 in LB and then grown aerobically with shaking at 37°C until OD_600_ reached 0.5. Cultures were untreated or treated with stress agents and incubated for 20 h with agitation at 37°C. Serial dilutions were performed in phosphate-buffered saline and plated on LB for colony-forming unit quantification after 24 h of incubation. Experiments were performed with six replicates. Treatment conditions are indicated in the respective figures.

### Disk diffusion assays

*C. violaceum* wild-type, mutant, and complemented strains grown overnight in LB were diluted to OD_600_ 1.0 in LB. Twenty microliters of each culture was embedded in 20 mL of LB agar. Wells were created on the plate, and 30 µL aliquot of 10 µg/mL SN, 10 mM H_2_O_2_, 80 mM ZnCl_2_, or 100 mM FeCl_3_ solutions was applied to individual wells. After incubation for 24 h at 37°C, halos of growth inhibition around each well were measured. Halo diameter was quantified using ImageJ software. Experiments were performed with three biological replicates.

### Siderophore production assay

Siderophores were detected using the universal chrome azurol S (CAS) agar plate assay (58) modified by replacing MM9 medium with peptone-sucrose agar (PSA) (26, 57). Ten microliters of *C. violaceum* cultures was spotted onto PSA-CAS agar plates, and siderophore production was evaluated by detection of orange halos that appeared after incubation for 24 h at 37°C.

### Co-transcription by RT-PCR

The *C. violaceum* wild-type strain was grown in LB to OD_600_ 4.0. Total RNA was extracted using Trizol reagent (Invitrogen) and purified with Direct-zol RNA Miniprep Plus (Zymo Research). RT-PCR was performed with the SuperScript III One-Step RT-PCR System with Platinum Taq High Fidelity DNA Polymerase (Invitrogen). One microgram of each RNA sample and specific primers (Table S2) that amplify regions from *cioR* to *cioA* (645 bp) and *cioR* to *cioB* (1528 bp) was used in the reactions. PCR using Taq DNA polymerase and the same primer sets was performed with genomic DNA (positive control) and RNA (negative control) as templates.

### Gene expression by RT-qPCR

*C. violaceum* wild-type, Δ*cioR*, and Δ*cioR* complemented strains were grown in LB to OD_600_ 1.0. Total RNA was extracted using TRIzol according to the manufacturer’s protocols. Five hundred nanograms of total RNA from each sample was converted to cDNA using the QuantiTect reverse transcription kit (Qiagen). Quantitative PCR (qPCR) reactions were performed and quantified in a Bio-Rad CFX96 (Bio-Rad), using SYBR Green master mix, specific primers (Table S2), and 2 µL of cDNA. Data from three biological replicates were normalized by an endogenous control (gene CV_3376) and a reference condition (WT in LB).

### Construction of transcriptional *lacZ* fusions and β-galactosidase assay

The region upstream of the *cioRAB* operon was amplified by PCR with designated primers (Table S2), cloned into the pGEM-T easy plasmid (Promega), and subcloned into the pRK*lacZ*290 vector to generate a transcriptional fusion to the *lacZ* gene. This construct was introduced into *C. violaceum* WT, ∆*cioR*, and ∆*cviR* strains. These strains were cultured in LB to OD_600_ 1.0 (LCD) or OD_600_ 4.0 (HCD). For stress conditions, the WT strain was grown in LB to OD_600_ 0.5, and cultures were untreated or treated for 30 and 60 min with stress agents as indicated in the respective figures. All cultures were assayed for β-galactosidase activity using a previously described protocol modified for *C. violaceum* (26, 40).

### Expression and purification of CioR

The coding region of *cioR* was PCR-amplified (Table S2) and cloned into the pET15b vector. The recombinant histidine-tagged protein (His-CioR) was overexpressed in *E. coli* BL21(DE3) by induction with 1 mM isopropyl-D-thiogalactopyranoside for 2 h at 37°C in LB. The His-CioR protein was purified from the soluble cell fraction using a 5 mL HisTrap HP column (Cytiva Life Sciences) on an AKTA Explorer FPLC system (Cytiva Life Sciences). Elution samples were concentrated and desalted by dialysis in storage buffer (20  mM Tris [pH 7.6], 150  mM NaCl, 0.1  mM EDTA, 5  mM DTT). Protein purification was checked by 15% SDS-PAGE.

### Electrophoretic mobility shift assay

A DNA 6-FAM-labeled probe containing the promoter region of the *cioRAB* operon was amplified by PCR (Table S2). DNA-binding reactions were performed in interaction buffer (20 mM Tris-HCl [pH 8.0], 50 mM NaCl, 1.5 mM MgSO_4_, 0.5 mM CaCl_2_, 0.1 mg/mL bovine serum albumin, 1 mM DTT, 0.05% NP-40, 10% glycerol), 0.1 mg/mL competitor salmon sperm DNA, 50 ng labeled 6-FAM DNA probe, and different concentrations of His-CioR in a final volume of 20 µL. All interaction reactions were incubated at 37°C for 25 min. Samples were separated by native 5% polyacrylamide gel electrophoresis in Tris-borate buffer. Competition assays were performed using 1 µM of His-CioR in the presence of a 10-fold excess of unlabeled specific (*cioRAB* promoter region) or nonspecific (CV_3376 coding region) probes. The fluorescence signal was captured by the Azure Sapphire Biomolecular Imager. The EMSA with stressors were performed using unlabeled DNA probe and detection by ethidium bromide staining.

## SUPPLEMENTAL MATERIAL

Supplemental material aem.02360-24-s0001.pdf Tables S1 and S2; Fig. S1.

ASM does not own the copyrights to Supplemental Material that may be linked to, or accessed through, an article. The authors have granted ASM a non-exclusive, world-wide license to publish the Supplemental Material files. Please contact the corresponding author directly for reuse.
