# Supplementary material for: A cytochrome bd repressed by a MarR family regulator confers resistance to metals, nitric oxide, sulfide, and cyanide in Chromobacterium violaceum
Source: Appl Environ Microbiol. 2025 Jan 24;91(2):e02360-24. doi: 10.1128/aem.02360-24 (PMC11837568; doi:10.1128/aem.02360-24)
Supplement: Supplemental material — Tables S1 and S2; Fig. S1. [file aem.02360-24-s0001.pdf]

Supplementary Material

Supplementary Table 1. Identification of transposon insertion sites in mutant strains screened for iron toxicity

Locus of T8 insertion	Function of gene product	No. of transposon hits	Mutant strain	Predicted size, aa	Insertion site, aa	MIC on LB $FeSO_4$ / $FeCl_3$ plates (mM)
---	---	---	WT	---	---	30 / 7
---	---	---	CVNAL	---	---	20 / 7
CV_0760	Two-component system, OmpR family	1	1E70	450	437	20 / 6
CV_1363	H-NS histone family protein	3	10F59, 10A92 and 3F91	101	39, 39 and 74	15 / 5
CV_1478	Mor domain-containing protein	1	5G53	143	128	20 / 6
CV_1867	Hypothetical protein	1	1A55	52	12	15 / 7
CV_2316	Probable transposase protein	1	1A70	263	IR	20 / 6
CV_2600	Two-component sensor/regulator (*csrA*)	1	5A53	924	532	20 / 6
**CV_3659**	**GbsR/MarR family transcriptional regulator**	**1**	**6B63**	**191**	**69**	**15 / 5.5**
CV_4019	UDP-N-acetyl-D-mannosamine dehydrogenase (*wecC*)	3	4B55, 6A74 and 9H61	413	168, 294 and 341	20 / 5.5
CV_4021	Glycosyltransferase family 4 protein	1	2D10	408	236	20 / 5.5
CV_4023	Peptidoglycan bridge formation glycyltransferase FemA/FemB family protein	1	4A73	331	31	30 / 5.5
CV_4028	Acylneuraminate cytidylyltransferase family protein	1	11G67	239	14	20 / 5.5
CV_4034	LegC family aminotransferase	2	3A13 and 11H91	383	82 and 308	15 / 5
CV_4035	SDR family NAD(P)-dependent oxidoreductase	3	12G25, 9D20 and 12C38	307	128, 128 and 151	20 / 5.5
CV_4129	Nucleotide sugar dehydrogenase	2	6B09 and 6B10	430	All in 114	20 / 6
CV_RS03115	Cupin fold metalloprotein, WbuC family	5	12H99, 10B101, 6E103, 10B103 and 9E94	169	All in 88	20 / 6

Supplementary Table 2. Oligonucleotides used in this work

Primer name	Sequence (5'→3')[a]	Description
**Identification of transposon insertion sites**		
CEKG2A	ggccacgcgtcgactagtacnnnnnnnnnnagag	Random primers used to identify
CEKG2B	ggccacgcgtcgactagtacnnnnnnnnnnacgcc	transposon insertion
CEKG2C	ggccacgcgtcgactagtacnnnnnnnnnngatat	
CEKG4	ggccacgcgtcgactagtac	Specific primers used to identify
lacZ-211	tgcgggcctcttcgctatta	transposon insertion
lacZ-148	gggtaacgccagggttttcc	
**Construction of mutant strains**		
CV_3659_del1	cctagcgggccccctgaacgtgctgcagctcg	*ApaI/HindIII* upstream flanking
CV_3659_del2	ggcctaaagcttcagaggtggaatattcatgac	fragment with 680 bp
CV_3659_del3	cctagcaagcttcagtcgctgtcccggctg	HindIII/SalI downstream flanking
CV_3659_del4	ggcctagtcgacggtcttcaaggccgccatc	fragment with 719 bp
CV_3658_del1[b]	cctagcgggccccgtgctgcatttcggcgag	*ApaI/HindIII* upstream flanking
CV_3658_del2[b]	ggcctaaagcttccaggccatcgcgatattg	fragment with 645 bp
CV_3657_del3	cctagcaagcttgccgacaagctcagctacc	*HindIII/BamHI* downstream
CV_3657_del4	ggcctaggatccttggcgtcgctgtaatagc	flanking fragment with 613 bp
CV_3657_RT_RV[b]	gaggtcgaagccgtccag	
**Construction of complemented strains**		
CV_3659_comp_FW	cctagcggatcccgctgggcgatctgctgg	*BamHI/EcoRI* 951 bp fragment
CV_3659_comp_RV	ggcctagaattcggccatcgcgatattgatgg	with CV_3659 and its promoter region
CV_3658_comp_FW[b]	cctagcggatccctgatgaagctgggctcgc	*BamHI/EcoRI* 2635 bp fragment
CV_3657_comp_RV	ggcctagaattcgctcgggacatcggcttc	with CV_3658 and CV_3657
**Heterologous expression**		
CV_3659_pET_FW	cctagccatatgaatattccacctctggtg	*Ndel/BamHI* 576 bp fragment
CV_3659_pET_RV	ggcctaggatcctcattctgcgtgctcccgg	with CV_3659 open reading frame
T7_promoter	taatacgactcactataggg	Sequencing of cloned insert.
T7_terminator	gctagttattgctcagcgg	
**β-galactosidase assay and EMSA**		
CV_3659_promot_FW	cctagcggatcccgctgggcgatctgctgg	*BamHI/HindIII* 298 bp fragment
CV_3659_promot_Rv	ggcctaaagcttcagaggtggaatattcatgac	with CV_3659 promoter region for pRK*lacZ*290 cloning
CV_3659_EMSA_FW	cgctgggcgatctgctgg	321 bp fragment with CV_3659
CV_3659_FAM_RV	6-FAM-aaatgcagcacgaaagcctgc	promoter region for EMSA
CV_3659_EMSA_RV	aaatgcagcacgaaagcctgc	
CV_3376_FW	aagccggcttcgactacatc	113 pb fragment of CV_3376
CV_3376_RV	cgacacttccggattggtga	coding region
**RT-qPCR**		
CV_3658_FW	gcctcaacacgttgaagcac	124 pb fragment of CV_3658
CV_3658_RV	tagtcgttgctgcgggtttc	coding region
CV_3657_FW	acaaaccgtggtggaacgc	118 pb fragment of CV_3657
CV_3657_RV	aacagccaggaagtggaggt	coding region
CV_3376_FW	aagccggcttcgactacatc	113 pb fragment of CV_3376
CV_3376_RV	cgacacttccggattggtga	coding region

[a]Restriction enzyme recognition sites are underlined.

[b] Primers also used in RT-PCR to check co-transcription of the *cio* genes.

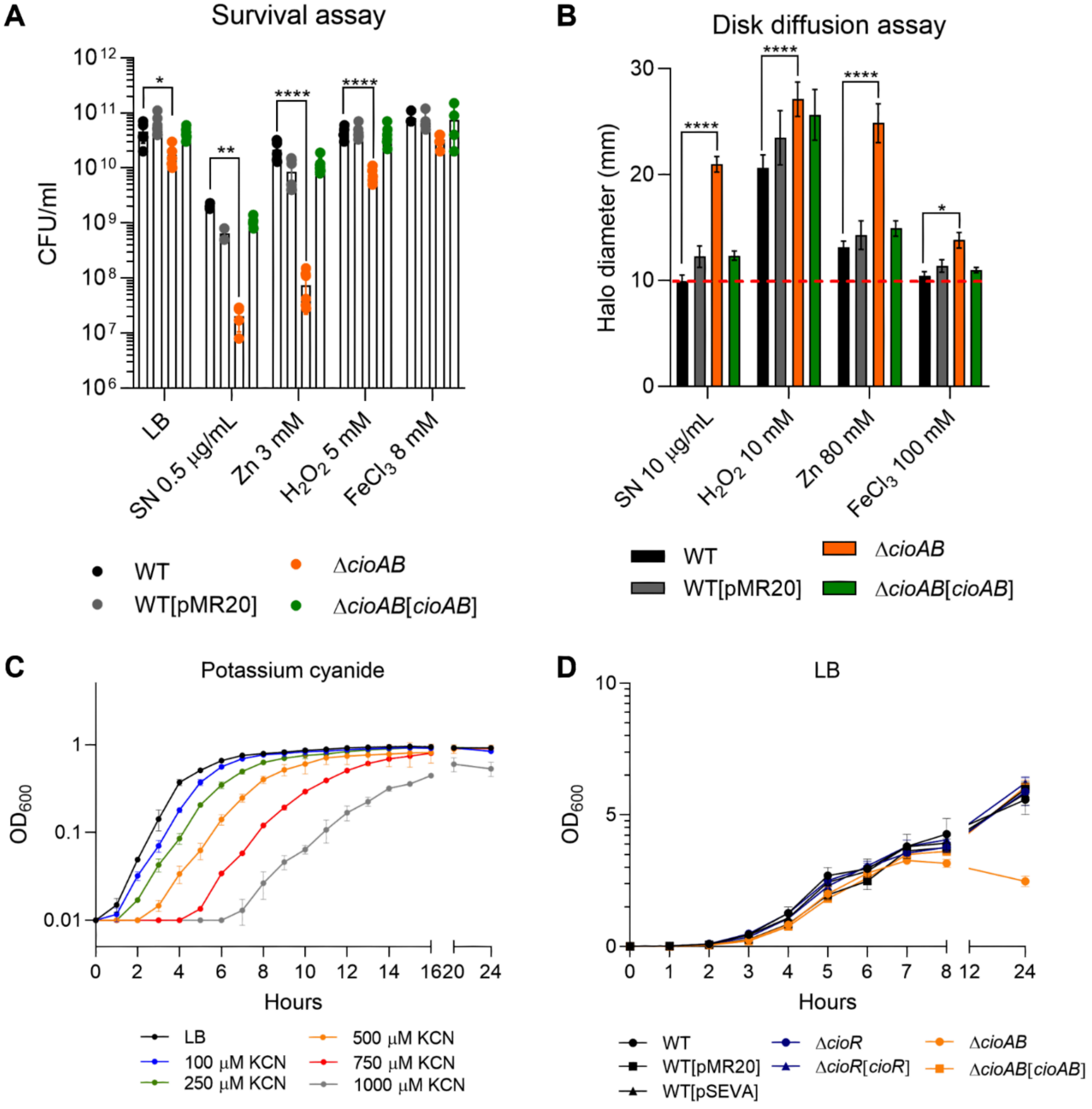

Supplementary Figure 1. Phenotypic tests of *C. violaceum* WT and *cio* mutants under various treatment conditions. A and B. The Δ*cioAB* mutant is susceptible to multiple stress conditions. **A.** Survival assays. Indicated strains were grown in LB until OD_{600} 0.5. The cultures were untreated or treated and incubated for 20 h under agitation at 37°C. Serial dilutions were plated on LB for CFU counting after 24 h of incubation. **B.** Disk diffusion assays. The strains were embedded in LB plates and 30 μL aliquots of

various compounds were added to wells. After incubation at 37°C for 24 h, halos of growth inhibition were measured. Data are from three biological replicates. ****$p < 0.0001$; ***$p < 0.001$; **$p < 0.01$; *$p < 0.05$; when not indicated, not significant. Significance was determined by two-way ANOVA followed by Tukey's multiple-comparison test. **C.** Susceptibility of *C. violaceum* to cyanide. Growth curves were obtained on a BioTek Epoch2 microplate reader. The wild-type strain was grown in LB without or with different concentrations of potassium cyanide in 96-well plates for 24 h at 37°C under agitation. **D.** Cultures of the Δ*cioAB* mutant reached a lower cell density after late-exponential growth phase. Growth curves were obtained by measurement of OD_{600} of the cultures during the first eight hours (intervals of 1 h) and at 24 h. Indicated strains were grown in LB medium in glass tubes at 37°C under agitation.
